# Infection pre-Ad26.COV2.S-vaccination primes greater class switching and reduced CXCR5 expression by SARS-CoV-2-specific memory B cells

**DOI:** 10.1038/s41541-023-00724-9

**Published:** 2023-08-12

**Authors:** Robert G. E. Krause, Thandeka Moyo-Gwete, Simone I. Richardson, Zanele Makhado, Nelia P. Manamela, Tandile Hermanus, Nonhlanhla N. Mkhize, Roanne Keeton, Ntombi Benede, Mathilda Mennen, Sango Skelem, Farina Karim, Khadija Khan, Catherine Riou, Ntobeko A. B. Ntusi, Ameena Goga, Glenda Gray, Willem Hanekom, Nigel Garrett, Linda-Gail Bekker, Andreas Groll, Alex Sigal, Penny L. Moore, Wendy A. Burgers, Alasdair Leslie

**Affiliations:** 1 Africa Health Research Institute, Durban, 4001 South Africa; 2 School of Laboratory Medicine and Medical Sciences, University of KwaZulu-Natal, Durban, 4001 South Africa; 3 National Institute for Communicable Diseases of the National Health Laboratory Services, Johannesburg, South Africa; 4 MRC Antibody Immunity Research Unit, School of Pathology, Faculty of Health Sciences, University of the Witwatersrand, Johannesburg, South Africa; 5 Institute of Infectious Disease and Molecular Medicine, University of Cape Town, Observatory, South Africa; 6 Division of Medical Virology, Department of Pathology, University of Cape Town, Observatory, South Africa; 7 Department of Medicine, University of Cape Town and Groote Schuur Hospital, Observatory, South Africa; 8 Wellcome Centre for Infectious Diseases Research in Africa, University of Cape Town, Observatory, South Africa; 9 Hatter Institute for Cardiovascular Research in Africa, Faculty of Health Sciences, University of Cape Town, Observatory, South Africa; 10 South African Medical Research Council, Cape Town, South Africa; 11 Division of Infection and Immunity, University College London, London, WC1E 6BT UK; 12 Centre for the AIDS Program of Research in South Africa, Durban, South Africa; 13 Discipline of Public Health Medicine, School of Nursing and Public Health, University of KwaZulu-Natal, Durban, South Africa; 14 Desmond Tutu HIV Centre, Cape Town, South Africa; 15 Department of Statistics, TU Dortmund University, Dortmund, Germany; 16 Max Planck Institute for Infection Biology, Berlin, 10117 Germany

**Keywords:** DNA vaccines, Viral infection

## Abstract

Neutralizing antibodies strongly correlate with protection for COVID-19 vaccines, but the corresponding memory B cells that form to protect against future infection are relatively understudied. Here we examine the effect of prior SARS-CoV-2 infection on the magnitude and phenotype of the memory B cell response to single dose Johnson and Johnson (Ad26.COV2.S) vaccination in South African health care workers. Participants were either naïve to SARS-CoV-2 or had been infected before vaccination. SARS-CoV-2-specific memory B-cells expand in response to Ad26.COV2.S and are maintained for the study duration (84 days) in all individuals. However, prior infection is associated with a greater frequency of these cells, a significant reduction in expression of the germinal center chemokine receptor CXCR5, and increased class switching. These B cell features correlated with neutralization and antibody-dependent cytotoxicity (ADCC) activity, and with the frequency of SARS-CoV-2 specific circulating T follicular helper cells (cTfh). Vaccination-induced effective neutralization of the D614G variant in both infected and naïve participants but boosted neutralizing antibodies against the Beta and Omicron variants only in participants with prior infection. In addition, the SARS-CoV-2 specific CD8+ T cell response correlated with increased memory B cell expression of the lung-homing receptor CXCR3, which was sustained in the previously infected group. Finally, although vaccination achieved equivalent B cell activation regardless of infection history, it was negatively impacted by age. These data show that phenotyping the response to vaccination can provide insight into the impact of prior infection on memory B cell homing, CSM, cTfh, and neutralization activity. These data can provide early signals to inform studies of vaccine boosting, durability, and co-morbidities.

## Introduction

The COVID-19 pandemic has caused more than 769 million infections and 6.9 million deaths (WHO dashboard: 11 August 2023). As global vaccination programs progress, vaccines are being administered to individuals with or without prior exposure to COVID-19. Therefore, it is important to understand the dynamics of the vaccine response in these two groups, as this could inform future vaccine dosing, scheduling and provide insight into homogeneous or heterogeneous vaccine boosting. In addition, it provides a unique opportunity to study immunological mechanisms that govern vaccine effectiveness and longevity and uncover potential early biomarkers of these essential metrics. All currently approved COVID-19 vaccines are based on the ancestral SARS-CoV-2 Wuhan-1 viral spike protein^1–3^, which facilitates viral entry into host cells via the host ACE2 and TMPRS22 surface receptors^4^. Eliciting antibodies against this spike and its receptor binding domain (RBD) can block viral adhesion and neutralize it, thereby preventing infection^5–9^. A functional antibody response, therefore, serves as an important correlate of protection for vaccine efficacy^10,11^ and includes Fc-dependent antibody effector functions^12^. However, as SARS-CoV-2 variants continue to emerge, the specificity and cross-reactivity of antibodies elicited through infection, vaccination, or the combination of both remain to be established as their affinity for and functionality against new variants may differ. This is to be expected since infection would prime responses to the whole virus and would depend on the infecting variant, whereas vaccines generally induce a spike-specific response, with most vaccines based on the ancestral virus.

Adenovirus vectored and mRNA vaccines targeting the viral spike have been rolled out worldwide, where the former is considerably cheaper to produce and distribute^13^. Following positive safety and immunogenicity data^3^, the single dose Ad26.COV2.S vaccine, manufactured by Johnson and Johnson, was distributed to healthcare workers as an early response to the pandemic in South Africa (SISONKE trial at clinicaltrials.gov NCT04838795). In this vaccine, the human Adenovirus 26 vector displays a stabilized form of the SARS-CoV-2 spike on its surface^1^. It induces functional antibody and T-cell responses against the vaccine strain that although reduced, cross-react with several SARS-CoV-2 variants^14–16^. Although the neutralizing antibody response following Ad26.COV2.S vaccination is three-to-five-fold lower than titers triggered by mRNA vaccines^14–18^, it showed an overall effectiveness of 82% against severe COVID-19^3^.

Functional antibodies, elicited by both infection and vaccination, are produced by antigen-specific memory B cells that have successfully matured into antibody-secreting cells which include both plasmablasts and plasma cells. These long-lived cells are a vital component for protection from future infection and severe disease^19,20^. Infection with SARS-CoV-2 elicits a memory B cell response to COVID-19, which matures 5–8 months after infection even though circulating antibodies may wane^21–24^. Studies of individuals receiving an mRNA vaccine found that prior infection profoundly boosted the specific B cell memory response^2,25^. Likewise, prior infection has been shown to boost SARS-CoV-2 specific antibody titers following vaccination^16,26–30^. However, the memory B cell response is induced by the single dose of Ad26.COV2.S vaccine has not been studied to date, nor has the impact of prior infection.

Here we tested samples collected as part of SISONKE, a phase 3B trial of Ad26.COV.2.S in South African health care workers^31^. COVID-19 seropositivity before vaccination within this sub-cohort was 56%^16^. We demonstrate that prior infection significantly increases the magnitude of the SARS-CoV-2-specific memory B cell response, its expression of the germinal center homing receptor CXCR5 and class switching following vaccination. This ultimately boosts the quantity and quality of the antibody response. We show strong associations between several B cell characteristics and the quality of the vaccine response, both before and after vaccination, particularly with CXCR5 expression and class switching. These were complemented with SARS-CoV-2-specific cTfh and CD8+ responses. Overall, these data provide insights into the boosting effects of prior infection on the B cell response to the single dose of Johnson and Johnson Ad26.COV2.S vaccination.

## Results

### Cohort description

To determine the impact of prior SARS-CoV-2 infection on the frequency and phenotype of the antigen-specific memory B cell response to the Johnson and Johnson Ad26.COV2.S vaccine, we studied 30 vaccinated participants, with known prior SARS-CoV-2 infection history, sampled before and after receiving a single dose of Ad26.COV2.S. Participants were grouped accordingly: naïve (for those with no history of infection) and infected (for those previously infected with SARS-CoV-2). All participants with an infection history were either asymptomatic or mildly symptomatic. Each participant’s COVID-19 history was confirmed by RT-PCR (*n* = 8) or serology (*n* = 14; Roche Elecsys® S and N test) or both (Supplementary Table 1). The infected group was further separated into those from the first wave (May–August 2020) when the D614G virus dominated, the second wave (November 2020–January 2021) when the Beta variant was dominant, and those for whom no data is available regarding the timing of infection or viral sequence (Supplementary Table 1). For all participants, peripheral blood mononuclear cells (PBMCs) were obtained prior to vaccination and 28 days post-vaccination. Although antigen-specific memory B cell responses can be detected as early as 7 days post-exposure, the 28-day time point was selected since the memory B cell populations developing in secondary lymphoid organs equilibrate with frequencies in the blood from 21 days after antigen exposure, thus reflecting the germinal center-derived memory response^25,32^. For a subset of participants, samples were also available from days 56 and 84 post-vaccination.

### Identifying SARS-CoV-2-specific memory B cells

**Fig. 1:**
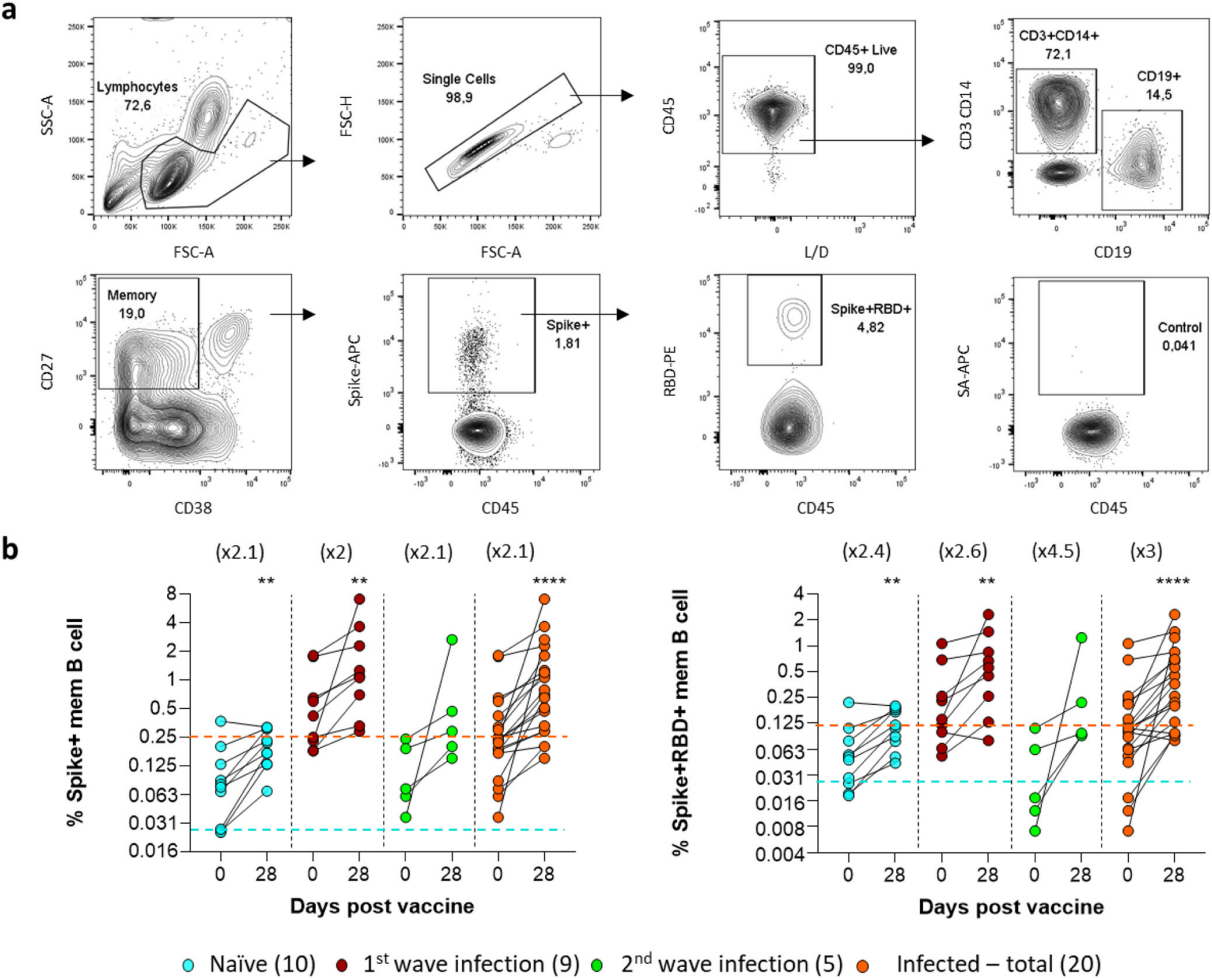
Spike and receptor binding domain (RBD)-specific memory B cell responses prior to (baseline) and 28 days post-vaccination. **a** Gating strategy identifying the CD19+ B cells (top row) and subsequent memory (CD27+), Spike+ (Spike-APC), and Spike+RBD+ (RBD-PE) memory B cells (bottom row). The spike and RBD baits were both from the D614G virus. The final panel serves as a background control for Streptavidin-APC (SA-APC). All population frequencies are relative to the gated parent population in the previous plot. A dump channel removed CD3 and CD14 positive cells. **b** The total Spike+ and Spike+RBD+ B cell memory responses were compared prior to (baseline) and at day 28 after vaccination. The fold increase of the response per group is indicated in brackets above each plot. The key indicates the COVID-19 infection history of vaccinated participants with sample numbers in brackets. The dashed lines represent the initial median response for the naïve (blue) and previously infected (orange) groups. Statistical analyses were performed using the Wilcoxon test for baseline and day 28 time points and Mann–Whitney for day 0 comparisons between groups. *P* values are denoted by *≤0.05; **<0.01; ***<0.001; and ****<0.0001.

SARS-CoV-2-specific B cells were identified using fluorochrome-labeled viral spike and receptor binding domain (RBD) proteins. SARS-CoV-2-specific memory B cells were identified by the expression of CD19, CD27 and binding either spike alone (Spike+), or spike and RBD together (Spike+RBD+; Fig. 1a). As expected, both the Spike+ and Spike+RBD+ populations were significantly expanded at day 28 post-vaccination (Fig. 1b). Notably, there was a significantly higher Spike+ and Spike+RBD+ memory B cell frequency prior to vaccination (day 0) in the infected group from the first wave of the epidemic relative to the naïve group (*P* = 0.0006, data not shown) and the second wave infections (*P* = 0.018, data not shown). The frequency of Spike+ and Spike+RBD+ memory B cells post-vaccination in naïve individuals was equivalent to the pre-vaccination levels of first-wave infected individuals. By contrast, individuals infected in the second wave with the Beta variant had similar pre and post-vaccine B cell responses to naïve individuals to spike alone; however, this group had the greatest fold increase of Spike+RBD+ memory B cells (4.5-fold). The baits used in this study were based on the D614G virus, and a concern was that Beta variant spike-specific B cells could have been underestimated. Pape et al. demonstrated substantial cross-reactivity using these reagents, however^25^. In addition, we successfully detected spike and RBD binding memory B cell responses from Beta variant infected participants using baits based on the D614G virus (Supplementary Fig. 1).

### Longitudinal memory B cell and antibody responses

**Fig. 2:**
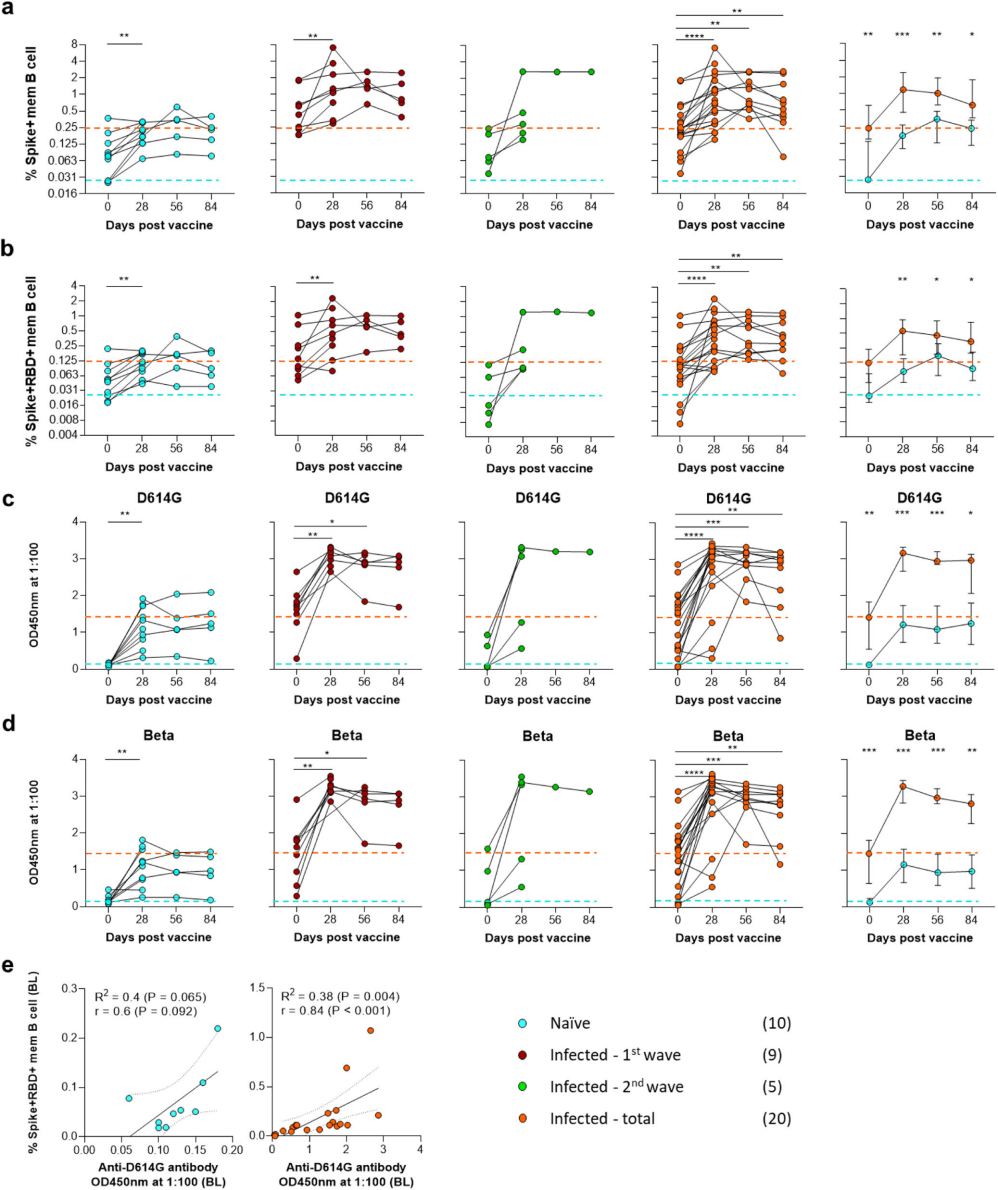
Longitudinal B cell memory and corresponding antibody responses to Spike and/or RBD. **a** The Spike+ and **b** Spike+RBD+ memory B cell responses were tracked over a 3-month longitudinal follow-up at 28-day intervals. Individual longitudinal plots are followed by median (±95% CI) longitudinal group plots. The dashed lines represent the initial median response for the naïve (blue) and previously infected (orange) groups. The corresponding longitudinal antibody responses are specific for the D614G spike in **c** and the Beta variant spike in **d** are shown in a similar layout. **e** Spearman non-parametric correlation of the Spike+RBD+B cell memory versus D614G spike-specific antibody response at baseline (BL). The key indicates the COVID-19 infection history of vaccinated participants with sample numbers in brackets. Statistical analyses were performed using the Wilcoxon test for longitudinal time points versus day 0 and Mann–Whitney test between naïve and previously infected groups. *P* values are denoted by *≤0.05; **<0.01; ***<0.001; and ****<0.0001.

In a subset of individuals with additional time points, we confirmed the Spike+ and Spike+RBD+ memory B cell responses persisted for three months in both the naïve and infected groups (Fig. 2a, b); with both showing a non-significant downward trend from day 28. The median frequency for the infected group was significantly greater than naïve individuals at all time points.

In parallel, we quantified SARS-CoV-2-specific antibodies in matched plasma samples and observed similar trends. As shown in different participants from the same cohort^16^, previous infection significantly boosted the total SARS-CoV-2 D614G and Beta binding antibodies (Fig. 2c, d). In naïve individuals, binding antibody titers also increased but, as with memory B cells, only reached the levels seen prior to vaccination in the infected group. Notably, these data indicate that the Ad26.COV2.S vaccine had a strong boosting effect on individuals previously infected with the Beta variant, despite the apparently minimal B cell cross-reactivity at baseline between first and second wave samples detected using D614G baits.

To determine the relationship between SARS-CoV-2 specific B cells and antibodies prior to vaccination, we correlated Spike+RBD+ memory B cell frequency and binding spike-specific antibody titer in baseline samples (Fig. 2e). There was a strong and highly significant correlation between baseline Spike+RBD+ memory B cell frequency and D614G-specific antibodies in previously infected individuals (R = 0.84; *P* < 0.001), confirming the expected relationship between these two parameters. In addition, although the antibody titers and B cell frequencies were much lower, a similar trend was observed in naïve individuals (*R* = 0.6; *P* = 0.09). Although this did not reach statistical significance, it suggests that low-frequency spike-cross-reactive B cells exist in individuals who have not been infected with SARS-CoV-2.

### Memory B cell class switching following vaccination

**Fig. 3:**
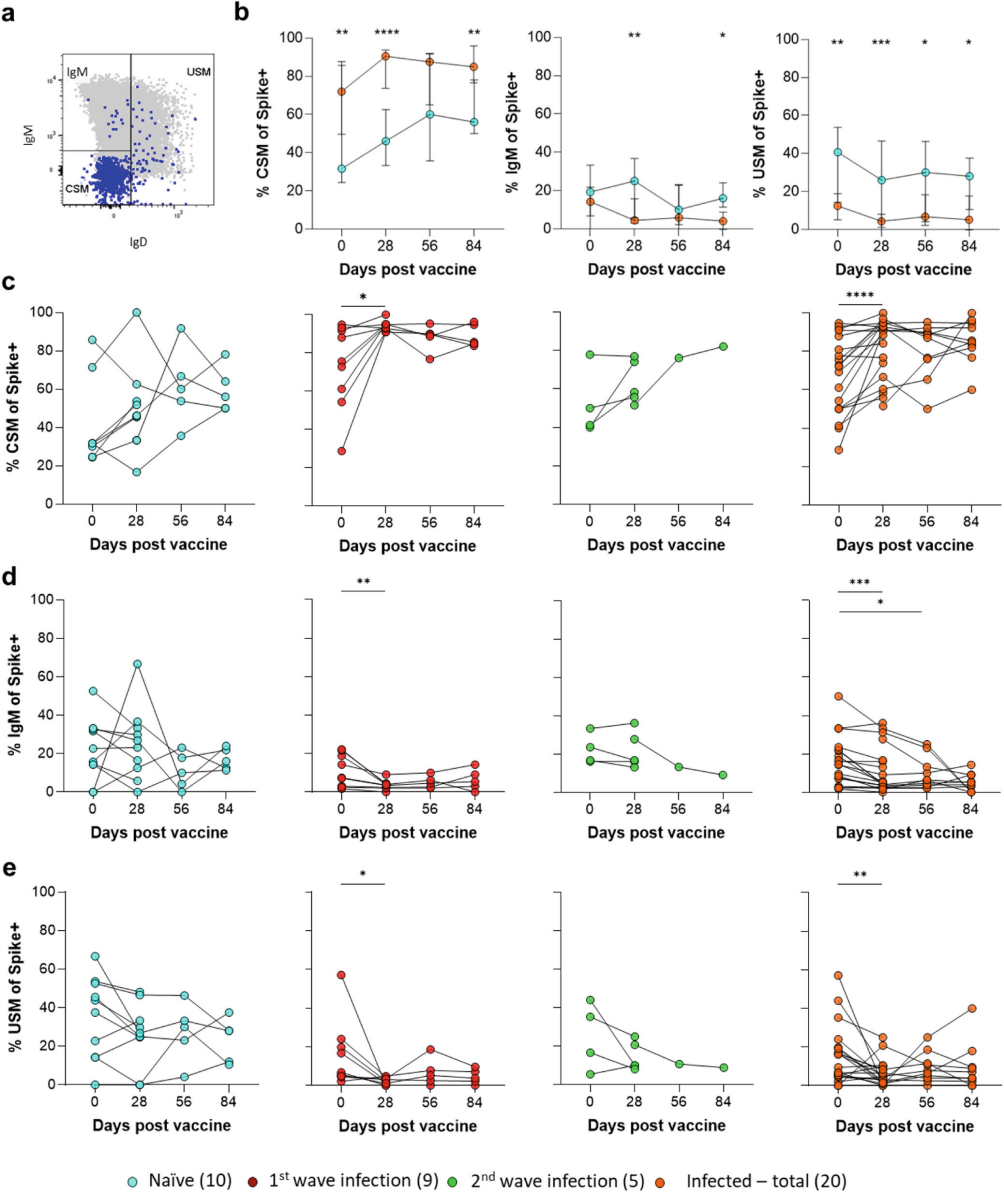
Longitudinal B cell memory class switching responses to Spike. **a** A representative Spike+ memory B cell population (blue) was overlaid onto the total memory B cell plot (gray) of IgM versus IgD for a naïve and infected vaccinee at day 28. The resulting IgM−IgD-class-switched memory (CSM), IgM+IgD-memory (IgM+) and unswitched memory (USM) responses were compared. **b** Spike+ memory B cell responses were tracked over a 3-month longitudinal follow-up at 28-day intervals. Longitudinal plots of median (±95% CI) CSM, IgM+, or USM are presented first allowing for comparison between naïve and infected groups. **c** This is followed by individual plots of CSM of naïve (blue), 1st wave infected (red), 2nd wave infected (green), and total previously infected (orange) participants. **d** Individual IgM+ and **e** individual USM responses. The key indicates the COVID-19 infection history of vaccinated participants with sample numbers in brackets. Statistical analyses were performed using the Mann–Whitney test between naïve and previously infected groups and the Wilcoxon test for longitudinal time points versus day 0. *P* values are denoted by *≤0.05; **<0.01; ***<0.001; and ****<0.0001.

To examine class switching, an important process in the maturation of the antibody response^33,34^, we examined surface expression of IgM and IgD on Spike+RBD+ memory B cells (Fig. 3a). At all timepoints, a greater proportion of these cells were fully class-switched (IgM− and IgD−) in infected individuals, except on day 56 (Fig. 3b). Both naïve and infected groups displayed increased class switching from day 0 to 84, consistent with a maturing B cell response (Fig. 3c). This is consistent with prior and continued B cell maturation in the infected group; while in naïve individuals they expressed more IgM (Fig. 3b, d), as expected for a primary response to antigen^35^. Unswitched IgD+Spike+RBD+ memory B cells were detectable in subjects with prior infection, suggesting a pool of naïve-specific B cells persists in these individuals (Fig. 3b, e). Overall, these data confirm that prior SARS-CoV-2 infection had a significant impact on the frequency of virus-specific B cell memory, binding antibody titer, and class switching.

### Activation and homing marker expression after vaccination

**Fig. 4:**
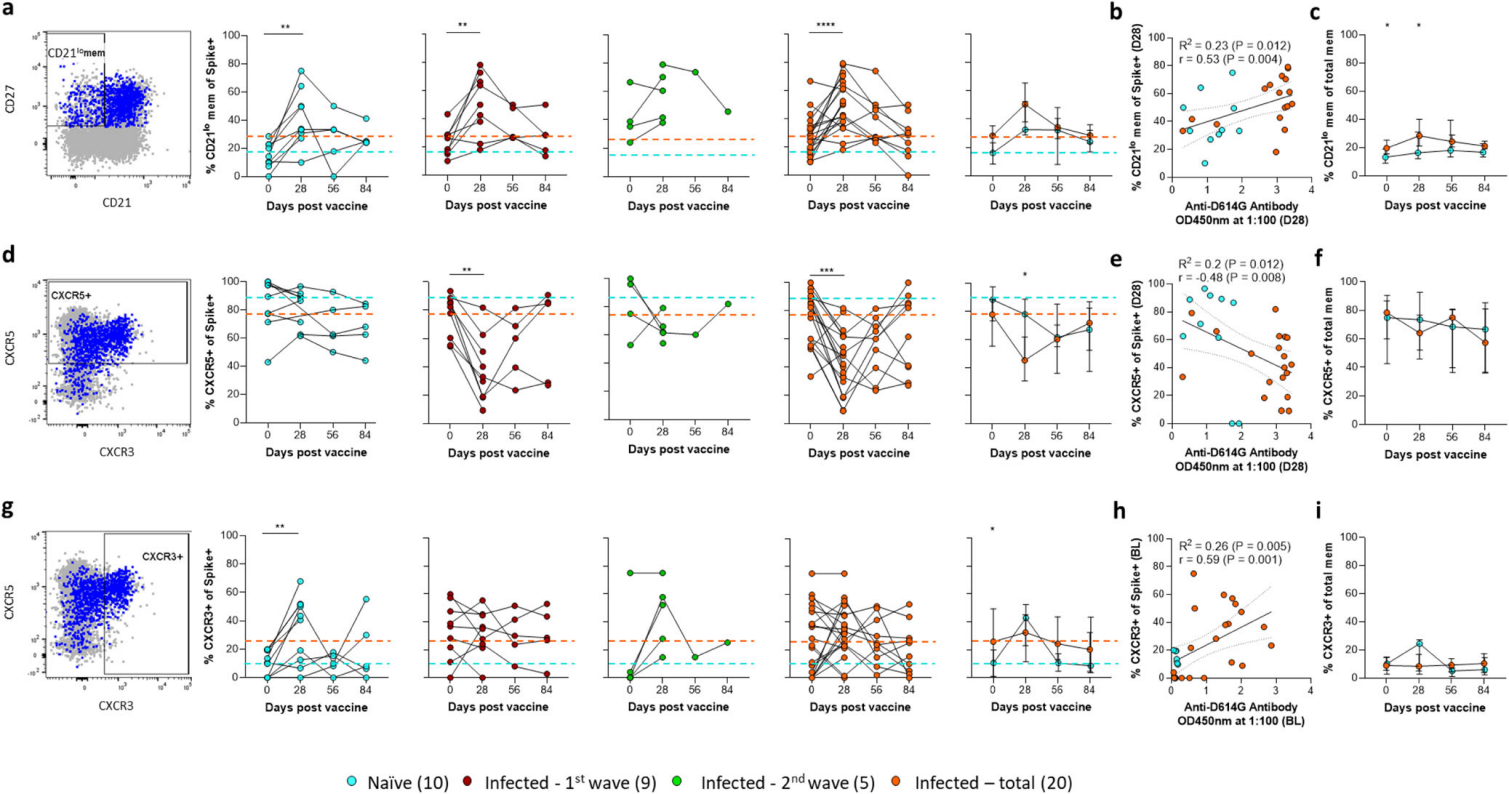
Longitudinal B cell activation and homing responses following vaccination. **a** The Spike+ memory B cell population (blue) was overlaid onto the total B cell population (gray). Plotting CD27 against CD21 identifies a CD27+CD21^lo^ activated memory B cell population. Individual longitudinal plots for the three patient group responses are followed by the median (±95% CI) longitudinal group responses. **b** Spearman non-parametric correlation of the day 28 Spike+ active B cell memory versus the day 28 D614G spike-specific antibody response. **c** The median total activated memory B cell responses were compared. The dashed lines represent the initial median response for the naïve (blue) and previously infected (orange) groups. A similar layout was followed in **d**–**f**, focusing on the CXCR5+ germinal center homing B cells and **g**–**i** the lung homing CXCR3+ B cells. The correlation in **h** was of the baseline (BL) CXCR3+ and antibody response. The key indicates the COVID-19 infection history of vaccinated participants with sample numbers in brackets. Statistical analyses were performed using the Wilcoxon test for longitudinal time points versus day 0 and Mann–Whitney test between naïve and previously infected groups. *P* values are denoted by *≤0.05; **<0.01; ***<0.001; and ****<0.0001.

Since infection induced a detectable spike-specific memory response, we were interested in assessing its impact on the activation and homing of these B cells following vaccination. The B cell activation profile was assessed by expression of CD21, which is downregulated upon B cell activation by cognate antigen^36–39^. Both the naïve and infected groups showed significantly increased Spike+ memory B cell activation at day 28 post-vaccination, which waned longitudinally (Fig. 4a). Although the initial extent and peak activation levels were greater in the infected compared to the naïve group, this was not significant. However, there was a significant positive correlation between the activation of Spike+ memory B cells and the magnitude of the spike-specific antibody response at day 28 (Fig. 4b). In addition, activation of total memory B cells was higher in individuals with prior infection (Fig. 4c), potentially indicating stimulation of additional non-bait reactive memory B cells in this group.

To examine B cell homing, we measured the expression of CXCR5, which is required for migration to germinal centers via its ligand CXCL13^40–42^. This revealed a striking reduction in CXCR5 expression at day 28 in the infected group (Fig. 4d), which rebounded at day 56 and 84, with a similar trend observed when comparing the median fluorescence intensity (MFI) of this marker (Supplementary Fig. 2a). This phenomenon was less pronounced and was delayed in naïve individuals, with CXCR5 expression reaching its lowest level at day 56. No significant differences in CXCL13 levels were observed in a subset of matched plasma samples, although there was a trend for increased CXCL13 in the previously infected group (Supplementary Fig. 2c). Overall, there was a significant negative correlation between CXCR5 expression levels on Spike+ memory B cells and antibody titer (Fig. 4e), which was not reflected by differences in CXCR5 expression on memory B cells in general (Fig. 4f).

In addition, we measured the expression levels of CXCR3, a marker known to facilitate homing to inflamed lung mucosae during infection via CXCL9 and CXCL10^39,43,44^. Prior to vaccination, we observed a striking difference in the expression of this marker on Spike+ memory B cells, which was elevated in approximately half of the individuals with previous infection but not in naïve individuals. Following vaccination, CXCR3 expression increased on Spike+ memory B cells in most individuals with low levels prior to vaccination, but not in individuals with elevated CXCR3 expression at this timepoint (Fig. 4g), a trend once again mirrored by the MFI of this marker (Supplementary Fig. 2b). The median CXCR3 response for the second wave infected participants had a similar peak response to the naïve group at day 28, but then retained similar CXCR3 expression to the previously infected group at later time points, although this only included a single participant (Fig. 4g). Overall, we observed a positive correlation between expression levels of CXCR3 and spike-specific antibody titer at baseline (Fig. 4h). Again, these differences appear to be limited in antigen specific B cells and are not reflected in the general memory B cell pool (Fig. 4i). Taken together, these data indicate that prior infection with SARS-CoV-2 results in phenotypic differences in the spike specific memory B cell response to vaccination.

### Spike-specific T cell responses following vaccination

**Fig. 5:**
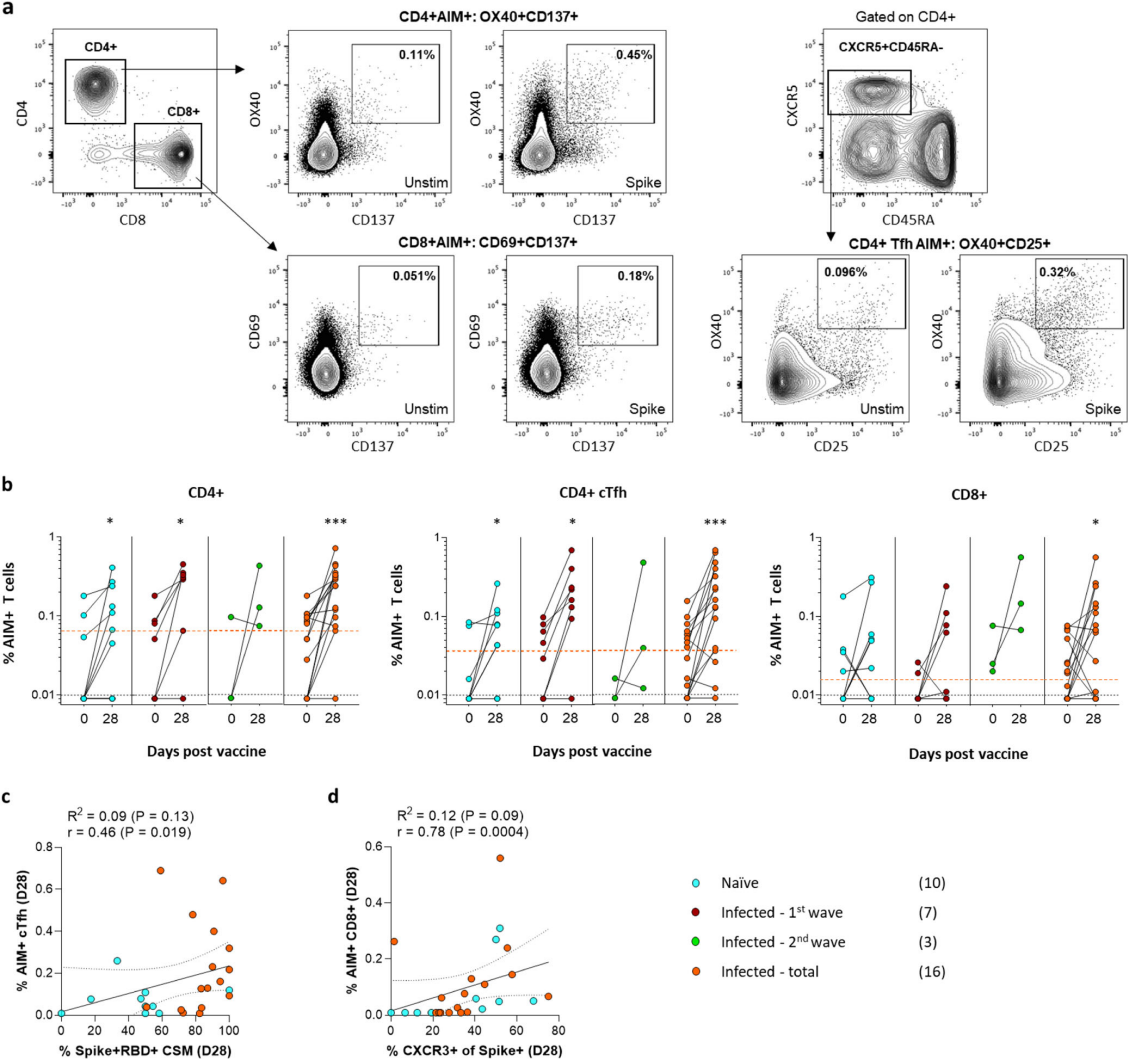
Spike-specific T cell AIM responses prior to (baseline) and 28 days post-vaccination. **a** Gating strategy identifying AIM+ T cells. Cells were first gated on live, CD3+ lymphocytes, and then total CD4+ or CD8+ T cells, with subsequent detection of CD4+ AIM+ T cells (OX40+ CD137; top row) and CD8+ AIM+ T cells (CD69+ CD137+; bottom row). To identify cTfh cells, the CXCR5+ CD45RA- CD4+ population was gated, with subsequent detection of AIM+ cells (OX40+ CD25+). Representative plots for an unstimulated (Unstim) and Spike peptide pool-stimulated (Spike) sample are shown. Frequencies of Spike-specific AIM+ cells are indicated in the gates. **b** Total Spike-specific CD4+, CD4+ cTfh, and CD8+ frequencies were compared prior to (baseline) and 28 days after vaccination, in naïve (blue) and previously infected (orange) groups. The dashed lines represent the assay cut-off (black) and initial median response for the previously infected (orange) group. The starting median for the naïve group fell on/below the assay cut-off. **c** Association between the frequency of AIM+ cTfh cells and Spike+ RBD+ CSM B cells at day 28. **d** Correlation between AIM+ CD8+ cells and CXCR3+ Spike+ memory B cells at day 28. The key indicates the COVID-19 infection history of vaccinated participants with sample numbers in brackets. Statistical comparisons were performed using the Wilcoxon test for day 0 and day 28-time points. *P* values are denoted by **<0.01 and ****<0.0001. Correlations were performed using the Spearman rank test.

Having observed changes in B cell activation and chemokine receptor expression, next we assessed the relationship between the vaccine-induced T cell response and memory B cells. T cells play an important role in vaccine protection, and CD4+ T follicular helper cells (Tfh) play an integral role in the germinal center response by providing help to B cells^45^. Using the activation-induced marker (AIM) assay, we measured the T-cell response to a peptide pool covering the full spike protein in 26 participants. The frequencies of spike-specific CD8+ and CD4+ T cells were determined using co-expression of CD69 and CD137 (for CD8+) and OX40 and CD137 (for CD4+)^46,47^. The circulating T follicular helper (cTfh) population was identified based on the expression of CXCR5 and CD45RA (i.e., CXCR5+ CD45RA−), and spike-specific cells were defined based on the co-expression of OX40 and CD25 (Fig. 5a). Vaccination induced spike-specific T cells in both naïve and previously infected individuals (Fig. 5b). We examined the relationship between cTfh, and memory B cell class switching (CSM) after vaccination and found a significant positive correlation between the frequencies of spike specific cTfh and Spike + RBD + CSM B cells at day 28 (Fig. 5c). Interestingly, we observed a significant correlation at day 28 between AIM+ CD8 + T cells and the Spike + CXCR3+ memory B cells (Fig. 5d). These data suggest that both CD4+ and CD8+ spike-specific responses may influence the B cell response to vaccination.

### Antibody functionality

**Fig. 6:**
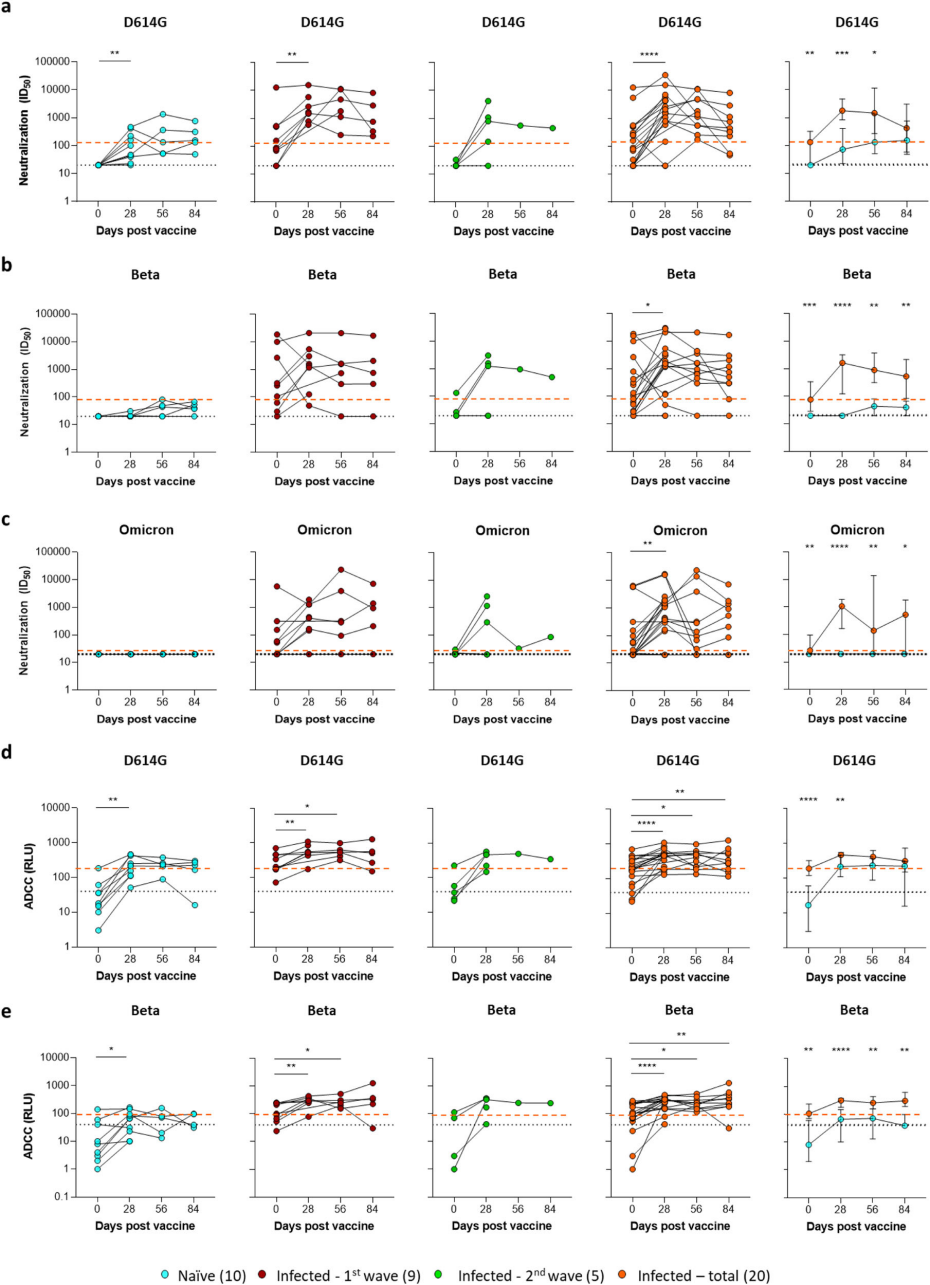
Longitudinal antibody-dependent viral neutralization and cellular cytotoxicity. **a–c** Viral neutralization ID_50_ titers versus the D614G, Beta, or Omicron variants respectively. Individual longitudinal plots are followed by a median (±95% CI) plot for comparison between naïve (blue) and previously infected (orange) groups. **d**, **e** Antibody-dependent cellular cytotoxicity (ADCC) represented as relative light units for participant plasma’s respective cross-linking of D614G or Beta spike-expressing cells and FcγRIIIa. The dashed lines represent the assay cut-off (black) and initial median response for the previously infected (orange) group. The starting median for the naïve group fell on/below the assay cut-off. The key indicates the COVID-19 infection history of vaccinated participants with sample numbers in brackets. Statistical analyses were performed using the Wilcoxon test for longitudinal time points versus day 0 and Mann–Whitney test between naïve and previously infected groups. *P* values are denoted by *≤0.05; **<0.01; ***<0.001; and ****<0.0001.

To determine how the observed difference in B cell phenotype related to functional antibody characteristics, we measured antibody neutralization using a pseudovirus-based assay (Fig. 6a–c) and antibody-dependent cellular cytotoxicity (ADCC; Fig. 6d, e) against the D614G strain (Fig. 6a, d), the Beta variant (Fig. 6b, e) and the Omicron variant (Fig. 6c). As expected, vaccination increased neutralization activity against D614G by day 28, which was significantly higher in individuals with prior infection. For the Beta and Omicron variants, however, this was only observed from individuals with prior infection, and neutralization activity in naïve individuals was ineffective against Omicron or remained low and never reached levels seen in previously infected subjects prior to vaccination. Consistent with our previous findings^16^, vaccination boosted ADCC activity against both viral variants irrespective of previous infection. Both neutralization and ADCC activity, however, were significantly higher against the Beta variant in previously infected individuals compared to naïve individuals at all timepoints.

### SARS-CoV-2-specific B cell, T cell, and antibody correlations

**Fig. 7:**
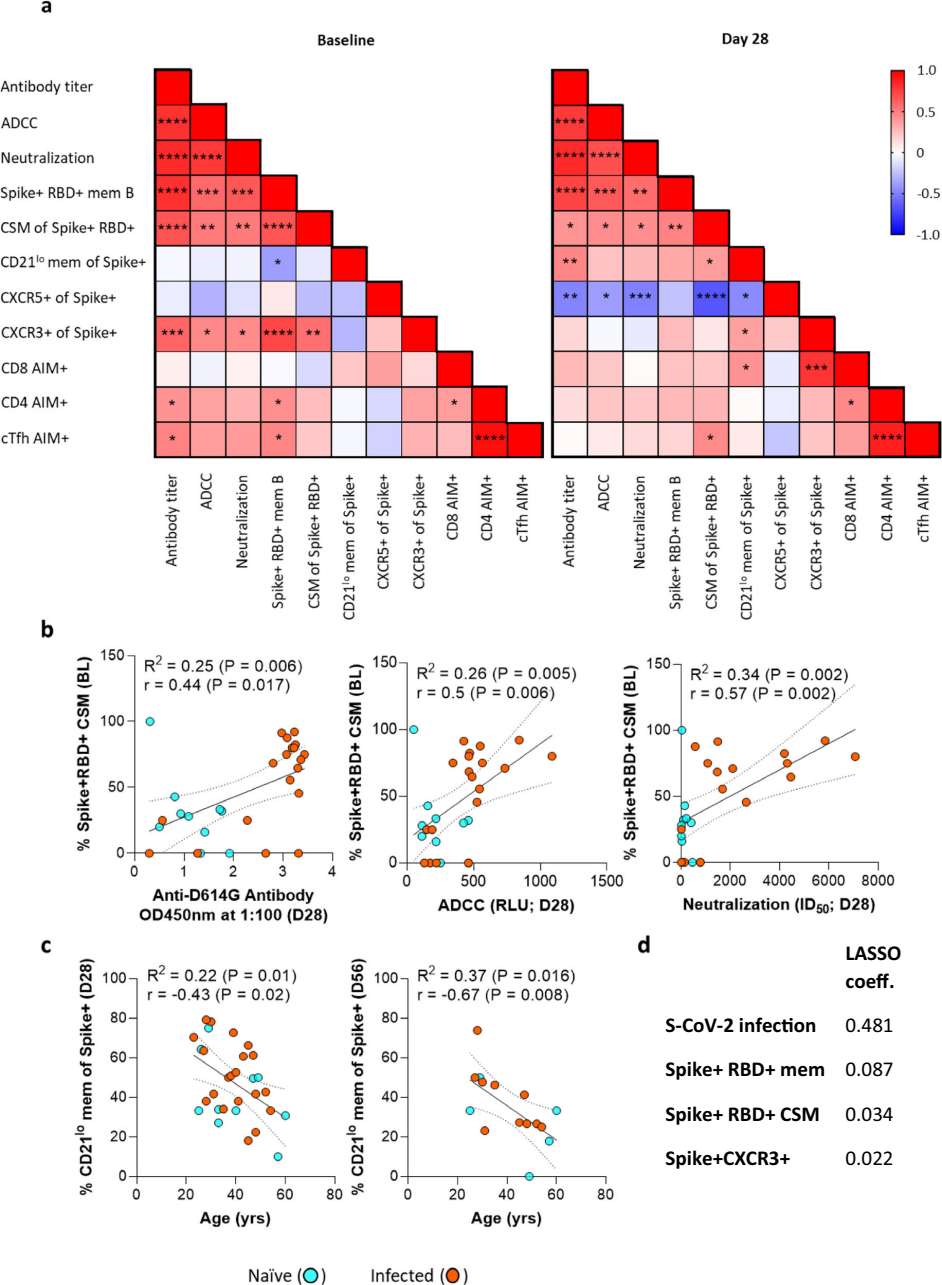
Correlations of the antibody, B and T cell responses following vaccination and the effect of age on B cell activation. **a** The Spearman rank correlation values (*r*) per comparison are presented as red (1.0) to blue (−1.0). Both naïve and previously infected participant data were included in the analyses (*N* = 24–30). **b** Baseline (BL) class-switched memory (CSM) and day 28 spike specific antibody, or day 28 ADCC, or day 28 neutralization were correlated. **c** The extent of Spike+ memory B cell activation was correlated with age for all participants. The respective *P* values denoted by *≤0.05; **<0.01; ***<0.001; and ****<0.0001. Antibody-dependent SARS-CoV-2 neutralization was selected as an outcome for LASSO regression analysis, with Spike+ memory B cell characteristics as covariates. The selected memory B cell predictors and their LASSO coefficient estimates were listed (intercept estimate was 4.338) in **d**, with the coefficient path plot and cross-validation (CV) error shown in Supplementary Fig. 3b, c).

Using a Spearman correlation analysis, we then examined the relationship between the phenotype of Spike+ memory B cells, SARS-CoV-2-specific antibody titer and functional activity, and AIM+ T cells (Fig. 7a) at baseline and post-vaccination (day 28). At baseline, we observed strong positive correlations between the total frequency of Spike+ memory B cells, degree of class switching, and CXCR3 expression and binding antibody titer and functional activity. The activation state and CXCR5 expression levels do not, however, correlate with antibody functionality at this timepoint. Following vaccination (day 28), the frequency of Spike+ memory B cells and degree of class switching remained correlated with these antibody characteristics, but the correlation with CXCR3 expression was lost. In addition, new associations were detected, including a positive correlation between activated spike-specific B cells and both binding titer and neutralization activity; and an inverse correlation between CXCR5 expression and binding titer, ADCC, and neutralization. On days 56 and 84, positive correlations were retained between spike-specific B cells and spike-binding antibodies, and notably day 56 presented the best correlation of CSM with neutralization (Supplementary Fig. 3a).

Furthermore, there is a strong and highly significant negative correlation between CXCR5 expression and the frequency of class switching on spike-specific memory B cells. This is consistent with the homing of memory B cells to germinal centers to undergo further class switching as well as memory B cells transitioning to plasmablasts^34,48,49^. The importance of class switching is further supported by the fact that the degree of class switching on spike-specific B cells at baseline has the strongest correlation with antibody titer, neutralization and ADCC at 28 days post-vaccination (Fig. 7b). Importantly, individuals with a low fraction of CSM spike specific B cells displayed low neutralization and ADCC activity irrespective of prior infection. In addition, there is a strong positive correlation between CXCR3 expression and class switching at baseline, suggesting an association with B cell activation (Fig. 7a).

As increasing age is a known risk factor in both COVID-19 disease severity^50^ and reduced vaccine responsiveness^2,17,51^, we examined the impact of participant age on the spike-specific B cell response to vaccination. Notably, we observed significant negative associations between age and the activation of Spike+ memory B cells at days 28 and 56 (Fig. 7c), suggesting an age-related decline in B cell stimulation following vaccination. Finally, ten Spike+ memory B cell parameters were included as predictor variables in the least absolute shrinkage and selection operator (LASSO) regression model. The strongest predictor for the response variable of neutralization activity was a prior infection, and an additional three B cell parameters were chosen as relevant. All three have a positive effect and are listed in decreasing order (Fig. 7d): Spike+RBD+ memory B cell frequency, the degree of class switching (Spike+CSM), expression levels of CXCR3 (Spike+CXCR3). The LASSO coefficient paths plot and optimal penalty parameter selection plot are shown in Supplementary Fig. 3b, c.

## Discussion

We assess the capacity of a single dose of Ad26.COV2.S vaccine to induce specific B cell memory responses in a cohort that includes previously SARS-CoV-2 infected and naïve participants. Vaccination resulted in a 2-3-fold expansion of spike and RBD-specific memory B cells that were durable for three months in both groups, like other vaccines investigated to date^2,25,39,52^. B cell memory in the naïve participants remained significantly reduced but eventually reached the baseline levels of the infected group. Notably, the magnitude of B cell response directly correlated with SARS-CoV-2 spike-specific antibody titer both prior to and following vaccination, validating the use of B cell baits to monitor memory B cell responses.

These data build on recent work showing that the Ad26.COV2.S vaccine elicits functional antibody and T cell responses to SARS-CoV-2 and emerging variants^14–16^. These studies did not, however, examine the memory B cell response. Previous work on mRNA SARS-CoV-2 vaccines also found differences in the magnitude of specific memory B cells induced in naïve and previously infected individuals. Interestingly, for these vaccines, the specific memory B cell levels observed in naïve individuals only reach the baseline level of the previously infected groups after the second dose, despite the antibody responses increasing as early as day 14^2,25,52^. By contrast, in this study, SARS-CoV-2-specific memory B cells reached this level in naïve individuals after a single dose of the Ad26.COV2.S vaccine. In addition, phenotyping of the SARS-CoV-2-specific memory B cell response revealed striking differences that correlated directly with antibody titer and functional activity and provides potential mechanistic insight into how prior infection changes the vaccine response.

Most studies investigating the humoral response to vaccination have focused more on the antibody characteristics rather than on the B cells generating them. However, the B cell memory compartment is vital for antibody recall responses as it is maintained for long periods after infection and may be called upon to: respond to secondary challenges; boost antibody titers in response to high viral load; and adapt to a heterologous secondary challenge, such as with variants of concern, during which memory may be drawn back into germinal centers for further affinity maturation and class switching^49,53^ or mature rapidly into antibody-secreting plasmablasts^49^. Therefore, understanding the type of B cell immune memory elicited by vaccination with/without prior infection can help inform how individuals may respond to subsequent infection or vaccination. Memory B cell responses to SARS-CoV-2 infection can persist for at least 5–8 months post-infection^21–24^. This is consistent with the observed positive correlation between the pre-vaccine frequency of SARS-CoV-2-specific memory B cells and antibody titers in the infected group. However, the fact that we observed a similar trend in the naïve group was somewhat unexpected. The antibody titer and spike-specific B cell frequency were low in these individuals and only just above background levels. However, because the two independent measures correlate, they likely reveal pre-existing cross-reactive memory B cells in these individuals. This may be explained by the conserved nature of spike proteins amongst other common coronaviruses^5^. In addition, SARS-CoV-2 binding naïve B cells have been observed in SARS-CoV-2-naïve individuals, suggesting that germline-encoded antibodies might have some cross-reactivity^25,54^. As new SARS-CoV-2 variants continue to emerge, the cross-reactivity of memory B cells is likely to be important in how prior infection and/or vaccination impact immunity.

Despite this potential cross-reactivity, the B cells we observed in naïve individuals had a significantly greater IgM memory phenotype than the infected group, which is a characteristic of a primary response to an antigen^35^. Additionally, CXCR3+ expression was extremely low compared to the infected group, consistent with data showing this marker is elevated following persistent antigen exposure^39^. Thus, it seems likely that both cross-reactive and de novo B cell responses emerged on vaccination. Going forward, phenotyping memory B cells in this way can help to tease apart the B cell response to subsequent SARS-CoV-2 variants and modified vaccines.

Although vaccination increased CXCR3 expression in naïve individuals, it returned to baseline levels by day 56. In previously infected individuals, however, a consistent fraction of spike-specific B cells expressed CXCR3 at all timepoints. This receptor facilitates homing to inflamed respiratory mucosae and its expression is IFN-γ-dependent involving the IFN-γ-induced transcription factor T-bet^39,43,44,55^. This suggests that memory B cells induced by prior infection may be better primed to home to the lung than those generated by single-dose vaccination. Importantly, there was a strong correlation between the AIM + CD8 + T cell and Spike+CXCR3+ memory B cell responses. Antigen-specific CD8 + T cells can either produce IFN-γ themselves or induce its production in CD4+ Tfh cells which then drives CXCR3 expression and class switching in germinal center B cells^44,56^. Vaccine boosting does increase B cell CXCR3 expression^39^ and although this has not been measured directly for COVID-19 vaccines, Pape et al.^25^ describe increased atypical memory B cells following mRNA vaccine boosting. Atypical B cells express T-bet and CXCR3^39,57,58^. Together these data suggest a potential mechanism through which booster vaccination could improve SARS-CoV-2-specific memory B cell homing to lung mucosae.

Another striking phenotypic difference in the B cell response to vaccination between the naïve and infected group concerns CXCR5 expression, which controls germinal center homing^40,41^. Importantly, expression levels of this marker were similar between groups at baseline, indicating that the rapid downregulation of CXCR5 on SARS-CoV-2-specific memory B cells in previously infected individuals was explicitly in response to vaccination. B cells reduce their CXCR5 expression upon encountering their cognate antigen^59^. We hypothesize that this phenomenon may reflect a combination of events. Firstly, the trafficking of CXCR5+ B cells to germinal centers or the release of CXCR5lo spike specific memory B cells from secondary lymphoid organs^25,32^. Recruitment of memory B-cells via CXCR5 is mediated by increased CXCL13 production by cells in the germinal center, and vaccination has been associated with increased CXCL13 levels in plasma^60,61^. In addition, plasma levels of CXCL13 have been directly linked with germinal center activity following HIV infection and vaccination^42^. We did observe a trend for increased CXCL13 in participants with prior infection, although this was not statistically significant. Longitudinal follow-up of participants receiving BNT162B2 also failed to observe a significant increase in CXCL13 after vaccination or between naïve and previously infected individuals, suggesting this association can be challenging to detect^62^. Secondly, SARS-CoV-2-specific memory B cells elicited during infection are activated after vaccination and rapidly mature to plasmablasts^49^ which is associated with downregulation CXCR5^63^. Irrespectively, the biological significance of CXCR5 downregulation is supported by the inverse correlation after vaccination with class-switching on SARS-CoV-2-specific memory B cells and both antibody neutralization and ADCC activity. In addition, the AIM+ CD4+ and cTfh correlated with memory B cell class switching, a critical interaction that has also been demonstrated in natural infection^45^. Vaccine efficacy is primarily based on neutralization activity^10,11,64^, and these data provide mechanistic insight into how a differential memory B cell response to vaccination leads to higher neutralization titers in individuals with prior exposure. It will be essential to determine how this response compares to booster doses given to naïve individuals.

Using a LASSO approach, we identified class switching as the strongest memory B cell predictor of the antibody response to vaccination. The importance of class switching is supported by the link between rapid class-switched antibody responses and a positive COVID-19 disease outcome in unvaccinated individuals^12^. Class-switched memory B cells are more adept at mounting a rapid antibody response upon subsequent infection or antigen exposure^25,65,66^. In line with this, we observed significant correlations between the degree of class-switching on SARS-CoV-2 memory B cells at baseline and the quantity and quality of the antibody response measured at days 28 and 56. This is consistent with increased class switching in previously infected participants observed with mRNA vaccines, especially following the first dose^2,25,52^. Therefore, prior exposure affords a significant advantage to the extent of class-switched memory B cells induced by single-dose vaccination. Again, it will be essential to test if boosting naïve individuals can achieve the same degree of class switching as natural infection.

Another informative phenotypic marker from this study was CD21, which was included to measure B cell activation following vaccination^36–39^. The clear spike in SARS-CoV-2-specific B cell activation on day 28 indicated that vaccination achieved similar stimulation in naïve and infected individuals. This is consistent with human influenza and yellow fever vaccination experiments that found a peak of CD21^lo^ antigen-specific B cells at day 14–28 post-vaccination^67,68^. These studies also found evidence that CD21^lo^ B cells were recent germinal center immigrants and preferentially developed into long-lived plasma cells. Therefore, the negative correlation between age and the frequency of CD21^lo^ B cells, irrespective of prior exposure, provides potential mechanistic explanations for reduced vaccine efficiency observed in older individuals. Sampling additional early timepoints following vaccination may provide further insights into the initial response kinetics of B cell activation and uncover the impact of known co-morbidities such as untreated HIV or diabetes^69^. In addition, investigating the presence of correlations between CD21 expression and B cell regulatory markers may provide novel opportunities to modulate B cell activation during vaccination.

A limitation of this study is that we used spike and RBD bait proteins based on the D614G virus only. Therefore, we may have missed memory B cell responses present in individuals infected with the Beta variant. However, since the vaccine is based on the ancestral virus, we believe this reagent was suitable for studying the vaccine response. Pape et al.^25^ found that 62–74% of the memory B cells specific to ancestral spike protein were cross-reactive to Beta variant spike following mRNA vaccination, and we also demonstrate the possibility of detecting spike and RBD-specific responses in Beta-infected participants. For six of the infected participants, the infection wave could not be confirmed. This may have introduced variability into the data as vaccination timing post-infection could affect the dynamics of the memory B cell response. Despite this, there were no obvious outliers in these infected participants that would have suggested an abnormally high or effective response as most had similar trends to the wave one infected participants. A second limitation is the low sample number (*n* = 30) especially when separating participants based on infection wave. The sample number used was limited and dependent on sample availability at the time of testing. Analysis of each infection wave was included to evaluate trends in the different groups but reduced the power significantly. For this reason, we included statistical analyses of naïve (*n* = 10) versus total infected (*n* = 20) and presented correlations in which the initial trends were maintained and are now significant. We are aware that these comparisons may be suboptimal and should be interpreted with this in mind. Larger studies to confirm these observations and the effects of prior exposure to different viral variants would be beneficial and these should also consider the effect of the second immunization dose, especially in individuals that remained uninfected/naïve at the time of immunization. Nonetheless, significant differences were observed between naïve (*n* = 10) and previously infected (*n* = 20) participants.

After vaccination, the antibody response in the naïve group was reactive to both the D614G and Beta variant spike proteins, facilitating similar binding and ADCC, but this did not translate to equally reactive neutralization capacity. Critically the antibody neutralization activity in the naïve group against the Omicron variant was null, highlighting the importance of investigating boosting efficacy in this group for protective responses to the Omicron variant.

By combining antibody and T cell measures with expanded memory B cell phenotyping that included activation and homing markers, we gain mechanistic insight into the post-vaccination memory B cell response. This response is significantly more robust in participants with a history of COVID-19 and was maintained for the study duration (84 days). Of note, we point out an overall reduction in memory B cell activation associated with age, regardless of infection history. It would be important to compare the long-term memory duration in these groups and to study the effectiveness of boosting regimens, both regarding the memory response but also efficacy against new variants and breakthrough infections.

## Methods

### Participants and longitudinal follow up

Our study was based on the SISONKE Phase 3b trial, in which healthcare workers were given a single dose of Johnson and Johnson Ad26.COV2.S between 17 February and 26 March 2021. Participants were recruited at Groote Schuur Hospital (Cape Town, Western Cape, South Africa), to form part of a longitudinal cohort of 400 healthcare workers. We included 30 participants from this cohort in this study and these included 10 naïve and 20 previously infected vaccinees. Of the previously infected, 9 were infected during the first wave (May–August 2020 with the D614G virus); five during the second wave (November 2020–January 2021 with the Beta variant) and six were infected but the infection timing could not be confirmed. Infection history was confirmed by laboratory PCR and/or serology testing. The participant demographic and clinical data were summarized in Supplementary Table 1. The study was approved by the University of Cape Town Human Research Ethics Committee (HREC 190/2020 and 209/2020), the University of the Witwatersrand Human Research Ethics Committee (Medical; no. M210429), the Biomedical Research Ethics Committee at the University of KwaZulu–Natal (reference BREC/00001275/2020). Written informed consent was obtained from all participants.

### Serology screening

Plasma samples were screened for antibodies specific to the SARS-CoV-2 spike and nucleocapsid (N) proteins. This was done using the Roche Elecsys® S and N test (Roche Diagnostics, GmbH). Samples were analyzed on a Cobas e801 instrument and a result ≥0.8 U/mL was considered positive in the S assay, and ≥1.0 U/mL positive in the N assay, according to the manufacturer’s instructions. The samples were also tested for the presence of CXCL13 as per the manufacturer’s instructions and a 1:1 sample dilution (human CXCL13/BLC/BCA-1 Quantikine ELISA, R&D Systems, cat. DCX130).

### Peripheral blood mononuclear cell (PBMC) isolation

All blood was collected into heparin tubes and processed within 4 h of collection. A Ficoll-Paque (Amersham Biosciences, Little Chalfont, UK) density gradient sedimentation was used to separate the PBMCs as per the manufacturer’s instructions. PBMCs were cryopreserved in freezing media containing 10% DMSO in heat-inactivated fetal bovine serum (FBS, Thermo Fischer Scientific) and stored in liquid nitrogen.

### SARS-CoV-2 spike and receptor binding domain (RBD) proteins

The SARS-CoV-2 D614G and Beta variant spike and receptor binding domain (RBD) proteins were recombinantly expressed in human embryonic kidney (HEK) 293F suspension cells. Following a 6-day culture at 37 °C, 70% humidity, and 10% CO_2_, the expressed viral proteins were purified on a nickel resin, followed by size exclusion chromatography, and stored at −80 °C until use.

### SARS-CoV-2 spike and RBD B cell bait staining and flow cytometry

Spike, RBD and HIV gp120 biotinylated bait proteins were labeled with Streptavidin-APC (SA-APC) or Streptavidin-PE (SA-PE) at a molar ratio of 4 (bait) to 1 (SA-APC or SA-PE) as described^2^. Frozen PBMCs were thawed and rested for 1 h in a 37 °C, 5% CO_2_ incubator. After resting a minimum of 5 × 10^6^–1 × 10^7^ cells were stained with a surface stain antibody mix (Supplementary Table 2, markers 1–12) for 20 min at room temperature, washed twice with 1 ml PBS and then stained with the SA-APC and SA-PE labeled bait proteins for an additional 30 min at room temperature. All bait proteins were used at a final 1:25 dilution following titration experiments displaying clear dose dependence of the spike and RBD double-positive B cell population frequency, with a little background for the FMO control (Supplementary Fig. 1). All antibody and bait staining mixes were prepared in 10% FCS in PBS using the dilution factors indicated in Supplementary Table 2, with a final volume of 25 µl per set of cells to be stained. The cells were washed twice with 1 ml PBS and finally suspended in 0.5 ml PBS, kept on ice, and acquired on a BD FACS Aria Fusion III, and data was analyzed using FlowJo version 9.9.6 (Tree Star).

### Activation-induced marker (AIM) assay

Cryopreserved PBMCs were thawed, washed, and rested in RPMI 1640 containing 10% heat-inactivated FCS for 4 h prior to stimulation. PBMCs were seeded in a 96-well V-bottom plate at ~1 × 10^6^ PBMCs per well and stimulated with a SARS-CoV-2 spike peptide pool (15mers overlapping by 10 amino acids) based on the full ancestral protein (Miltenyi, 1 µg/mL in distilled water). As a negative control, PBMCs were stimulated with media alone. After 16 h of stimulation, cells were washed, stained with LIVE/DEAD™ Fixable Near Infrared Stain (Invitrogen, Carlsbad, CA, USA), and subsequently stained with the surface marker antibodies (Supplementary Table 3). Finally, cells were washed and fixed in CellFix (BD Biosciences). Samples were acquired on a BD LSRII flow cytometer and analyzed using FlowJo (v10, FlowJo, Ashland, OR, USA). Cells were gated on singlets, lymphocytes, live CD3+ cells, and CD4+ or CD8+ T cells. AIM+ cells were those co-expressing OX40 and CD137 (for CD4+) or CD69 and CD137 (for CD8+), while the AIM+ cTfh population was defined as CXCR5+ CD45RA− CD4+ T cells co-expressing OX40 and CD25. Results are expressed as the frequency of AIM+ CD4+ or CD8+ T cells, and AIM+ cTfh is expressed as the frequency of total CD4+ T cells. AIM+ responses presented are background subtracted (from the frequency of AIM+ cells in the unstimulated sample), and the threshold for AIM positivity was defined as >1.5× the unstimulated sample and >0.01%.

### Spike-specific enzyme-linked immunosorbent assay (ELISA)

96-well ELISA plates were coated overnight at 4 °C with viral spike protein at 2 µg/ml. Plates were blocked with 5% skimmed milk powder in PBS with 0.05% Tween 20. All plasma was diluted 1:100 in blocking buffer and added to the plate, after which the plates were incubated with secondary antibody at 1:3000 in blocking buffer. The signal was developed with a TMB substrate (Thermo Fischer Scientific) and 1 M H_2_SO_4_ was used to stop the reaction. Absorbance was measured at 450 nm. All assays included monoclonal antibodies CR3022 and BD23 as positive controls and palivizumab as a negative control.

### Pseudovirus neutralization assay

Two SARS-CoV-2 pseudotyped lentiviruses were prepared by co-transfecting a HEK293T cell line with either the D614G virus spike or the Beta spike (L18F, D80A, D215G, K417N, E484K, N501Y, D614G, A701V, 242-244 del) plasmids and a firefly luciferase lentivirus backbone plasmid. Participant plasmas were heat inactivated and incubated with each of the pseudotyped SARS-CoV-2 viruses for 1 h at 37 °C, 5% CO_2_. Following this incubation, the pseudoviruses were incubated with 1 × 10^4^ HEK293T cells overexpressing the ACE-2 surface receptor for 72 h at 37 °C, 5% CO_2_. Any cells that were successfully infected by the pseudoviruses, were detected by luminometry of the luciferase gene. The CB6 monoclonal antibody was used as a positive control for neutralization.

### Antibody-dependent cellular cytotoxicity (ADCC)

An indirect measure of ADCC was performed by assessing the ability of participant plasma to crosslink the FcγRIIIa (CD16) surface receptor of Jurkat-Lucia^TM^ NFAT-CD16 cells and SARS-CoV-2 spike expressing HEK293T cells. The HEK293T cells were transfected with 5 µg of SARS-CoV-2 D614G virus or Beta variant (L18F, D80A, D215G, K417N, E484K, N501Y, D614G, A701V, 242-244 del) spike plasmids using PEI-MAX 40000 (Polysciences) and incubated for 2 days at 37 °C, 5% CO_2_. Spike expression was confirmed by flow cytometry, using anti-spike CR3022 or P2B-2F6 and anti-IgG-APC staining. Heat-inactivated plasma was diluted 1:100 in R10 media (RPMI 1640 media with 10% FCS and 1% Penicillin/Streptomycin (Gibco, Gaithersburg, MD)). Control antibodies were used at 100 µg/ml and 1 × 10^5^ spike transfected HEK293T cells/well, of a 96-well culture plate and were incubated with the antibody-treated media for 1 h at 37 °C, 5% CO_2_. The Jurkat-Lucia^TM^ NFAT-CD16 cells (Invivogen) were added at 2 × 10^5^ cells/well and incubated for a further 24 h. A 20 µl aliquot of the culture supernatant was transferred to a white 96-well plate containing 50 µl/well QUANTI-Luc secreted luciferase and read immediately using a Victor 3 luminometer set to a 1 sec integration time. A no antibody control was used to subtract background luminescence. The monoclonal antibodies: Palivizumab served as a negative control, CR3022 as a positive control, and P2B-2F6 to differentiate the Beta from the D614G variant. A 1x cell stimulation cocktail (Thermo Fischer Scientific, Oslo, Norway) and 2 µg/ml ionomycin in R10 media were added and served as a positive control by inducing the transgene.

### Statistical analysis

All analyses were performed in Prism (v9; GraphPad Software Inc., San Diego, CA, USA), except for the LASSO analysis, which was performed in the statistical software R^70^. Nonparametric tests were used throughout, with Mann–Whitney and Wilcoxon tests used for unmatched and paired samples, respectively. All correlations reported are nonparametric Spearman rank correlations. *P* values < 0.05 were considered statistically significant and denoted by *≤0.05; **<0.01; ***<0.001; and ****<0.0001. The influence of several potential Spike+ memory B cell predictor variables (*p* = 10) on the SARS-CoV-2 neutralization was assessed by the so-called least absolute shrinkage and selection operator (LASSO) principle^71^, based on scaled covariates, and fitting a log-linear model via the glmnet R package^72,73^. For this method, the optimal tuning parameter *λ*, which reflects the amount of penalization and, hence, controls variable selection, is determined via 10-fold cross-validation based on the model’s deviance (see Supplementary Fig. 3b, c).

### Reporting summary

Further information on research design is available in the Nature Research Reporting Summary linked to this article.

### Supplementary information

Supplemental Information

Reporting Summary

## Data Availability

All data supporting the findings of this study are available within the paper and its Supplementary Information.
