# Supplementary material for: Infection pre-Ad26.COV2.S-vaccination primes greater class switching and reduced CXCR5 expression by SARS-CoV-2-specific memory B cells
Source: NPJ Vaccines. 2023 Aug 12;8:119. doi: 10.1038/s41541-023-00724-9 (PMC10423246; doi:10.1038/s41541-023-00724-9)
Supplement: Supplementary file 1 — Supplemental Information [file 41541_2023_724_MOESM1_ESM.pdf]

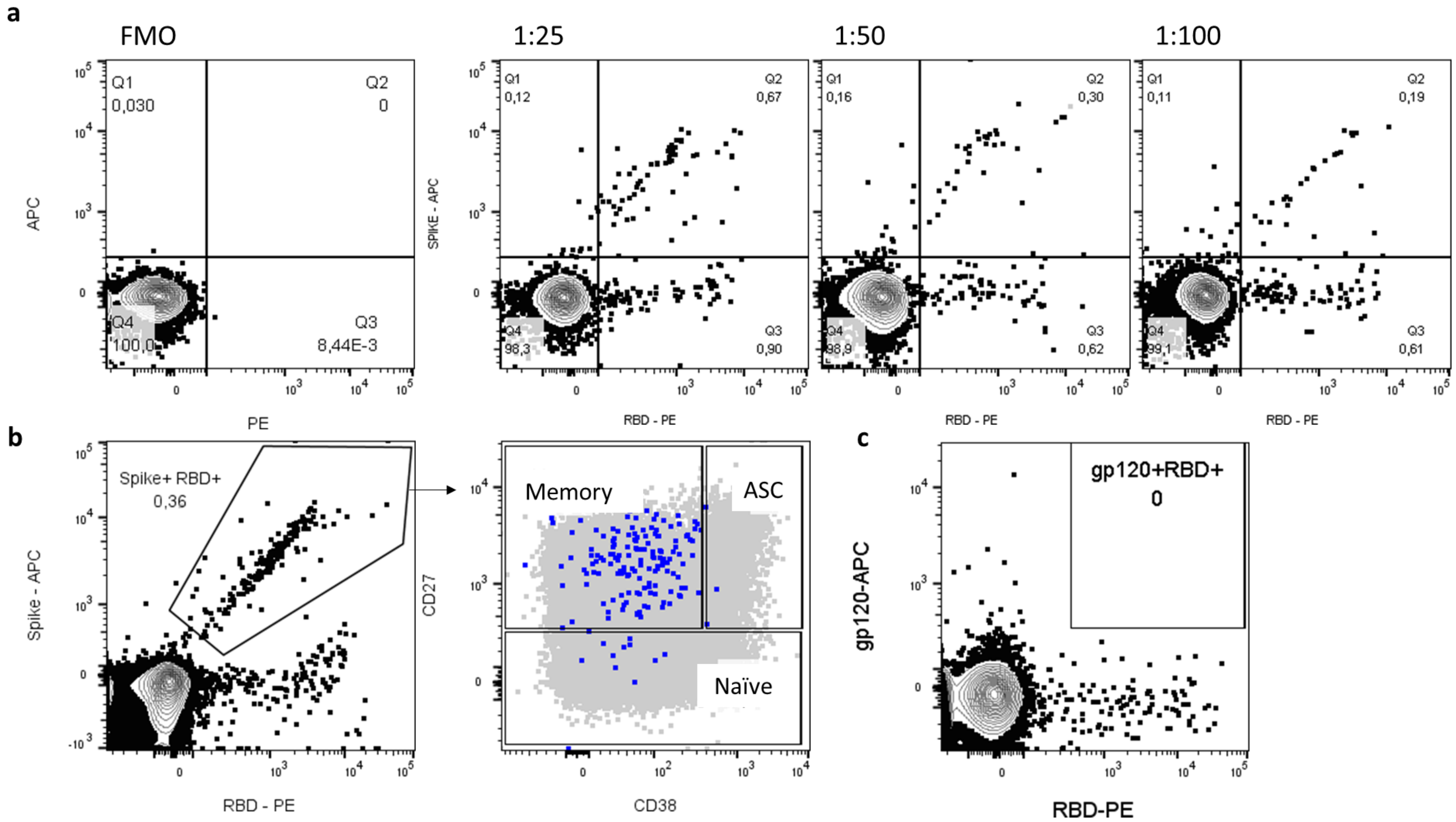

Supplementary Figure 1

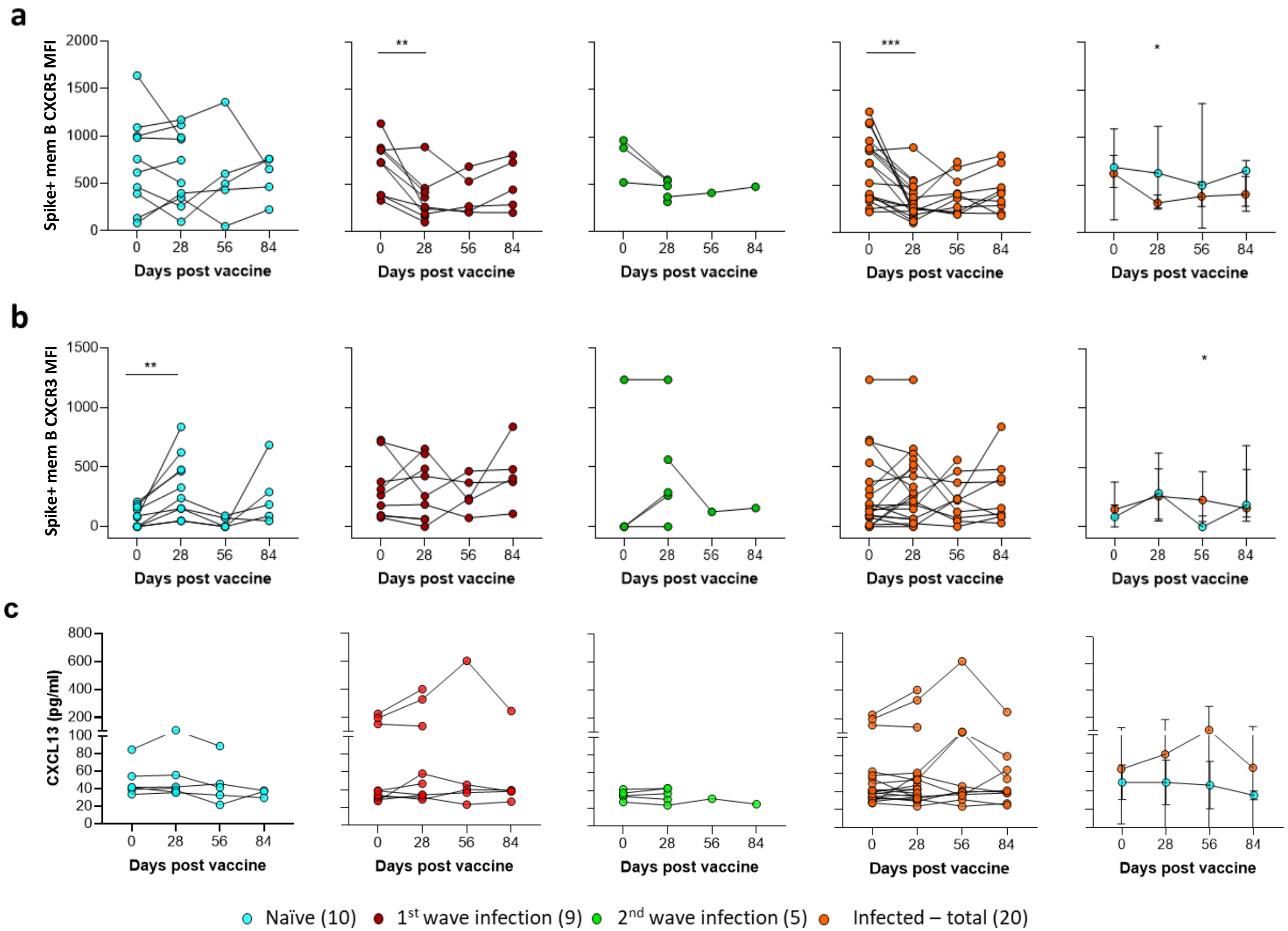

Supplementary Figure 2

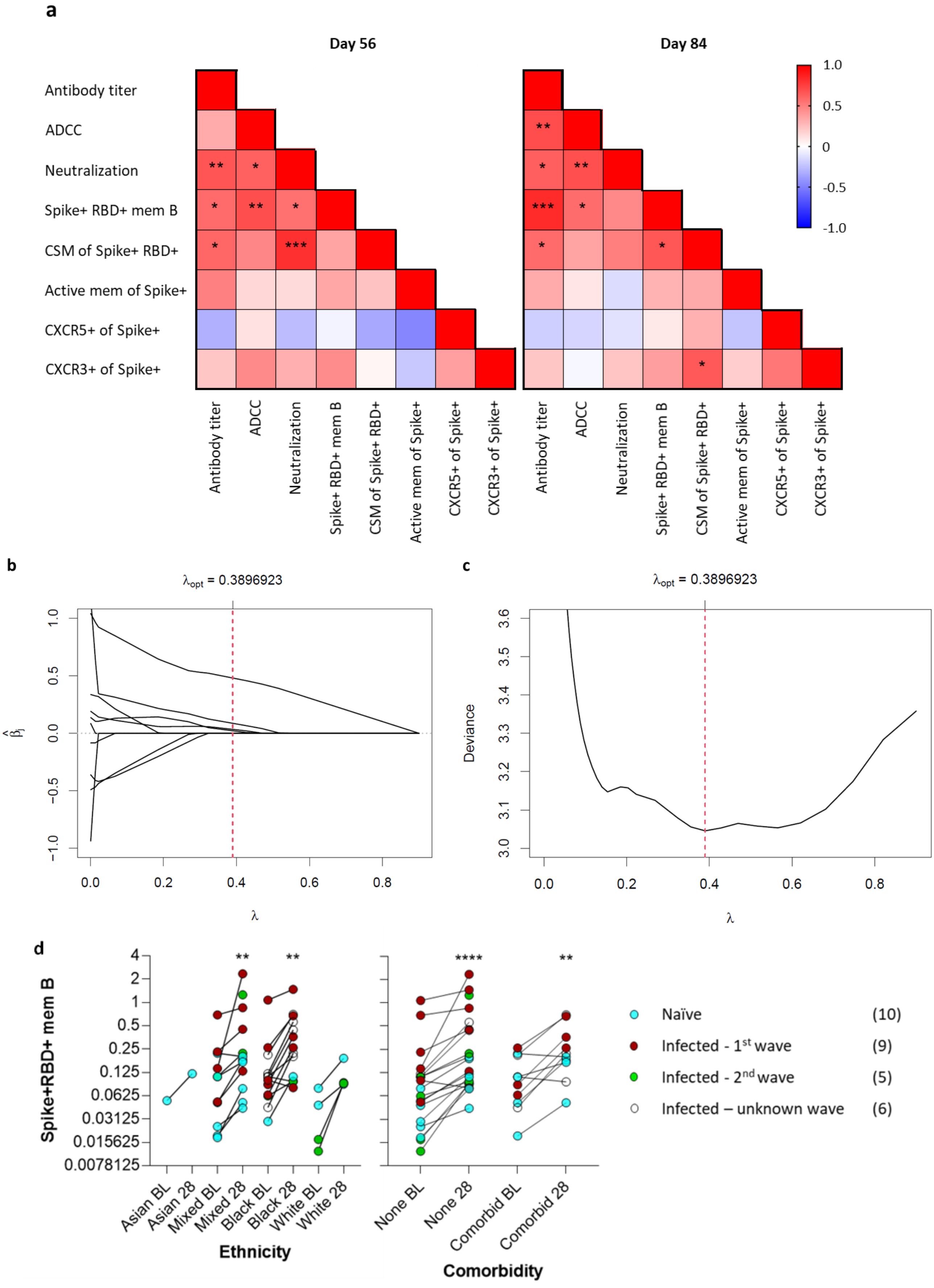

Supplementary Figure 3

Supplementary Table 1: Participant demographic and clinical data

	No previous infection	1st wave infection	2nd wave infection	Unconfirmed wave infection	TOTAL
N	10	9	5	6	30
**Demographic**					
Age (years)	36 [28-51]	39 [29-47]	41 [26-45]	39 [34-49]	39 [30-47]
Gender M:F	6M:4F	1M:8F	2M:3F	1M:5F	10M:20F
Ethnicity					
Black	1 (10%)	5 (56%)	1 (20)	6 (100%)	13 (43%)
White	2 (20%)	0 (0%)	2 (40%)	0 (0%)	4 (13%)
Mixed	6 (60%)	4 (44%)	2 (40%)	0 (0%)	12 (40%)
Other	1 (10%)	0 (0%)	0 (0%)	0 (0%)	1 (3%)
**Clinical**					
PCR SARS-CoV-2+	0 (0%)	8 (89%)	0 (0%)	0 (0%)	8 (40%)*
Serology SARS-CoV-2+	0 (0%)	9 (100%)	5 (100%)	6 (100%)	20 (100%)*
Days after vaccination					
28	31 [26-34]	29 [28-31]	30 [0]	30 [24-33]	30 [28-33]
56	67 [61-68]	60 [59-66]	56 [0]	58 [54-61]	60 [58-67]
84	92 [87-95]	88 [84-93]	84 [0]	84 [81-86]	87 [84-93]
Comorbidities					
Asthma	2 (20%)	1 (11%)	0 (0%)	0 (0%)	3 (10%)
Hypertension	2 (20%)	3 (33%)	0 (0%)	1 (17%)	6 (20%)
Obesity	1 (10%)	0 (0%)	0 (0%)	1 (17%)	2 (7%)
Diabetes mellitus	1 (10%)	0 (0%)	0 (0%)	0 (0%)	1 (3%)
HIV	0 (0%)	0 (0%)	0 (0%)	2 (33%)	2 (7%)
Other	1 (10%)	0 (0%)	0 (0%)	0 (0%)	1 (3%)
None	6 (60%)	6 (67%)	5 (100%)	3 (50%)	20 (67%)
>1 comorbidity	2 (20%)	1 (11%)	0 (0%)	1 (17%)	4 (13%)

The median age and interquartile range are stated per group. Gender is stated as (M) male and (F) female. * Percentages were calculated from total infected (n=20). Time of follow up visits is shown as median days and interquartile range following vaccination. HIV positive participants were on effective HAART.

Supplementary Table 2: B cell phenotyping panel

#	Marker	Lable	Clone	cat no	origin	Dilution	Supplier
1	L/D	APC-Cy7		L10119		1 in 200	Invitrogen
2	CD45	Hv500	H130	560777	mouse	1 in 50	BD Horizon
3	CD3	Bv711	OKT3	317328	mouse	1 in 50	BioLegend
	CD14	Bv711	M5E2	301838	mouse	1 in 25	BioLegend
4	CD19	Bv605	HIB19	302244	mouse	1 in 50	BioLegend
5	CD27	PE-Cy5	1A4CD27	6607107	mouse	1 in 25	Beckman Coulter
6	CD38	PECy7	HIT2	303516	mouse	1 in 50	BioLegend
7	IgM	PerCP/Cy5.5	MHM-88	314512	mouse	1 in 100	BioLegend
8	IgD	AF700	IA6-2	348230	mouse	1 in 25	BioLegend
9	CXCR5	AF488 (FITC)	RF8B2	558112	rat	1 in 100	BD Pharmingen
10	CXCR3	PE-CF594	IC6/CXCR3	562451	mouse	1 in 25	BD Horizon
11	CD21	Bv421	B-ly4	562966	mouse	1 in 100	BD Horizon
12	BAIT	SA-APC		405207		1 in 25	BioLegend
13	BIAT	SA-PE		405204		1 in 25	BioLegend
14	CD95 (Fas)	Bv650	DX2	305642	mouse	1 in 50	BioLegend

Supplementary Table 3: T cell phenotyping and AIM assay panel

#	Marker	Fluor	clone	cat no	Dilution	Supplier
1	L/D	APC-Cy7		L10119	1 in 2000	Invitrogen
2	CD4	ECD	T4	6604727	1 in 100	Beckman Coulter
3	CD3	Bv650	OKT3	317324	1 in 100	BioLegend
4	CD8	Bv510	RPA-T8	301048	1 in 100	BioLegend
5	CD45RA	Bv570	HI100	304132	1 in 100	BioLegend
6	CXCR5	Bv785	J252D4	356936	1 in 25	BioLegend
7	OX40	PECy7	ACT35	350012	1 in 50	BioLegend
8	CD25	PE	BC96	302606	1 in 33	BioLegend
9	CD69	FITC	FN50	310904	1 in 50	BioLegend
10	CD137	APC	4B4-1	309810	1 in 50	BioLegend

Supplementary Fig. 1: Reactivity and specificity of the wild type (D614G) SARS-CoV-2 Spike and RBD baits with SARS-CoV-2 infected patient B cells. a, Total B cells were used in titration experiments to determine the optimal dilution of spike and RBD bait proteins to use. A fluorescence minus one (FMO) control served to set the quadrant gates. **b,** Reactivity of total B cells from a HIV negative, Beta-variant infected patient with the wild-type (D614G) derived spike and RBD bait proteins. The bait-positive gated B cell population was overlaid onto the total B cell plot of CD27 versus CD38. **c**, Reactivity of memory B cells with the HIV antigen gp120 for an HIV positive patient versus the SARS-CoV-2 RBD bait protein.

Supplementary Fig. 2: MFI of homing markers CXCR5 and CXCR3 on spike+ RBD+ memory B cells and plasma levels of CXCL13. a, Surface expression levels of CXCR5 and **b**, CXCR3 measured as MFI for spike+ RBD+ memory B cell populations. **c**, Plasma levels of the CXCR5 ligand, CXCL13, were measured by ELISA and the overall mean ($^{+}/_{-}$ SD) were compared between groups. The key indicates the COVID-19 infection history of vaccinated participants with sample number in brackets.

Supplementary Fig. 3: Correlations of the antibody, B and T cell responses following vaccination and the LASSO analysis coefficient paths and optimal penalty parameter. a, The Spearman rank correlation values (r) per comparison are presented as red (1.0) to blue (-1.0) for day 56 and 84 comparisons. Both naïve and previously infected participant data was included in the analyses (N = 13-26). The respective *P* values denoted by * ≤ 0.05; ** < 0.01; *** < 0.001 and **** < 0.0001. **b**, The LASSO regression analysis coefficient path plot resulting after antibody dependent SARS-CoV-2 neutralization was selected as the outcome and the Spike+ memory B cell parameters were included as covariates (intercept estimate was 4.338). **c**, The plot for determination of the optimal LASSO penalty parameter, showing the course of the error versus penalty strength lambda in a 10-fold cross validation approach. **d**, The effect of ethnicity and underlying comorbidity on the spike and RBD specific memory B cell responses were assessed. The key indicates the COVID-19 infection history of vaccinated participants with sample number in brackets. Statistical analyses were performed using the Wilcoxon test between time points versus day 0 and Mann-Whitney test between naïve and previously infected groups. *P* values are denoted by * ≤ 0.05; ** < 0.01; *** < 0.001 and **** < 0.0001.
