# Supplementary material for: Infection pre-Ad26.COV2.S-vaccination primes greater class switching and reduced CXCR5 expression by SARS-CoV-2-specific memory B cells
Source: NPJ Vaccines. 2023 Aug 12;8:119. doi: 10.1038/s41541-023-00724-9 (PMC10423246; doi:10.1038/s41541-023-00724-9)
Supplement: Supplementary file 2 — Reporting Summary [file 41541_2023_724_MOESM2_ESM.pdf]

nature portfolio

Corresponding author(s): Al Leslie

Last updated by author(s): Jul 14, 2023

Reporting Summary

Nature Portfolio wishes to improve the reproducibility of the work that we publish. This form provides structure for consistency and transparency in reporting. For further information on Nature Portfolio policies, see our Editorial Policies and the Editorial Policy Checklist.

Statistics

For all statistical analyses, confirm that the following items are present in the figure legend, table legend, main text, or Methods section.

n/a	Confirmed	
☐	☒	The exact sample size (n) for each experimental group/condition, given as a discrete number and unit of measurement
☐	☒	A statement on whether measurements were taken from distinct samples or whether the same sample was measured repeatedly
☐	☒	The statistical test(s) used AND whether they are one- or two-sided<br>*Only common tests should be described solely by name; describe more complex techniques in the Methods section.*
☐	☒	A description of all covariates tested
☐	☒	A description of any assumptions or corrections, such as tests of normality and adjustment for multiple comparisons
☐	☒	A full description of the statistical parameters including central tendency (e.g. means) or other basic estimates (e.g. regression coefficient) AND variation (e.g. standard deviation) or associated estimates of uncertainty (e.g. confidence intervals)
☐	☒	For null hypothesis testing, the test statistic (e.g. F, t, r) with confidence intervals, effect sizes, degrees of freedom and P value noted<br>*Give P values as exact values whenever suitable.*
☒	☐	For Bayesian analysis, information on the choice of priors and Markov chain Monte Carlo settings
☐	☒	For hierarchical and complex designs, identification of the appropriate level for tests and full reporting of outcomes
☒	☐	Estimates of effect sizes (e.g. Cohen's d, Pearson's r), indicating how they were calculated

Our web collection on statistics for biologists contains articles on many of the points above.

Software and code

Policy information about availability of computer code

Data collection	No software was used.
Data analysis	FACSDiva 9 (BD Biosciences)<br>FlowJo version 9.9.6 (Tree Star)<br>GraphPad Prism Software version 9<br>glmnet R package version 4.1-3

For manuscripts utilizing custom algorithms or software that are central to the research but not yet described in published literature, software must be made available to editors and reviewers. We strongly encourage code deposition in a community repository (e.g. GitHub). See the Nature Portfolio guidelines for submitting code & software for further information.

Data

Policy information about availability of data

All manuscripts must include a data availability statement. This statement should provide the following information, where applicable:

- Accession codes, unique identifiers, or web links for publicly available datasets
- A description of any restrictions on data availability
- For clinical datasets or third party data, please ensure that the statement adheres to our policy

All data referred to in the manuscript is available in the manuscript or Supplementary files.

Field-specific reporting

Please select the one below that is the best fit for your research. If you are not sure, read the appropriate sections before making your selection.

☒ Life sciences ☐ Behavioural & social sciences ☐ Ecological, evolutionary & environmental sciences

For a reference copy of the document with all sections, see nature.com/documents/nr-reporting-summary-flat.pdf

Life sciences study design

All studies must disclose on these points even when the disclosure is negative.

Sample size	Samples included 10 COVID naive and 20 participants previously infected with COVID-19. These sample sizes were sufficient to identify statistically significant variation between the two participant groups.
Data exclusions	No data were excluded.
Replication	All assays were reproducible and included appropriate positive and/or negative controls to validate the assay performance.
Randomization	Participant samples were allocated into COVID naive or previously infected groups after B cell assays were concluded.
Blinding	Investigators performing B cell assays were blinded to the initial COVID status of the participants.

Reporting for specific materials, systems and methods

We require information from authors about some types of materials, experimental systems and methods used in many studies. Here, indicate whether each material, system or method listed is relevant to your study. If you are not sure if a list item applies to your research, read the appropriate section before selecting a response.

Materials & experimental systems

n/a	Involved in the study	
☐	☒	Antibodies
☐	☒	Eukaryotic cell lines
☒	☐	Palaeontology and archaeology
☒	☐	Animals and other organisms
☐	☒	Human research participants
☐	☒	Clinical data
☒	☐	Dual use research of concern

Methods

n/a	Involved in the study	
☒	☐	ChIP-seq
☐	☒	Flow cytometry
☒	☐	MRI-based neuroimaging

Antibodies

Antibodies used

NearIR Live/Dead stain, cat# L10119, Invitrogen
CD45 Hv500, clone HI30, cat# 560777, BD Horizon
CD3 Bv711, clone OKT3, cat# 317328, BioLegend
CD14 Bv711, clone M5E2, cat# 301838, BioLegend
CD19 Bv605, clone HIB19, cat# 302244, BioLegend
CD27 PE-Cy5, clone 1A4CD27, cat# 6607107, Beckman Coulter
CD38 PECy7, clone HIT2, cat# 303516, BioLegend
IgM PerCP/Cy5.5, clone MHM-88, cat# 314512, BioLegend
IgD AF700, clone IA6-2, cat# 348230, BioLegend
CXCR5 AF488, clone RF8B2, cat# 558112, BD Pharmingen
CXCR3 PE-CF594, clone IC6/CXCR3, cat# 562451, BD Horizon
CD21 Bv421, clone B-ly4, cat# 562966, BD Horizon
SA-APC, cat# 405207, BioLegend
SA-PE, cat# 405204, BioLegend
CD4 ECD, clone T4, ct# 6604727, Beckman Coulter
CD3 Bv650, clone OKT3, cat# 317324, BioLegend
CD8 Bv510, clone RPA-T8, cat# 301048, BioLegend
CD45RA Bv570, clone HI100, cat# 304132, BioLegend
CXCR5 Bv785, clone J252D4, cat# 356936, BioLegend
OX40 PECy7, clone ACT35, cat# 350012, BioLegend
CD25 PE, clone BC96, cat# 302606, BioLegend
CD69 FITC, clone FN50, cat# 310904, BioLegend
CD137 APC, clone 4B4-1, cat# 309810 BioLegend

Validation	All antibodies were validated for the species and application by the relevant manufacturer.

Eukaryotic cell lines

Policy information about cell lines

Cell line source(s)	Human Embryonic Kidney (HEK) 293F suspension cells from Dr Nicole Doria-Rose, VRC, USA<br>HEK293T cell line from Dr Michael Farzan, Scripps, USA<br>Jurkat-LuciaTM NFAT-CD16 cells (Invivogen, Cat # jktl-nfat-cd16)
Authentication	All cell lines were authenticated by the supplier.
Mycoplasma contamination	All cell lines tested negative for mycoplasma contamination.
Commonly misidentified lines<br>(See ICLAC register)	None.

Human research participants

Policy information about studies involving human research participants

Population characteristics	Covariates included prior COVID-19 infection, age and co-morbidities.
Recruitment	Our study was based on the SISONKE Phase 3b trial, in which health care workers were given single dose Johnson and Johnson Ad26.COV2.S between 17 February and 26 March 2021. Participants were recruited at Groote Schuur Hospital (Cape Town, Western Cape, South Africa), to form part of a longitudinal cohort of 400 health care workers. We included 30 participants from this cohort in this study and these included 10 naïve and 20 previously infected vaccinees.
Ethics oversight	The study was approved by the University of Cape Town Human Research Ethics Committee (HREC 190/2020 and 209/2020), the University of the Witwatersrand Human Research Ethics Committee (Medical; no M210429). Written informed consent was obtained from all participants.

Note that full information on the approval of the study protocol must also be provided in the manuscript.

Clinical data

Policy information about clinical studies

All manuscripts should comply with the ICMJE guidelines for publication of clinical research and a completed CONSORT checklist must be included with all submissions.

Clinical trial registration	clinicaltrials.gov NCT04838795
Study protocol	clinicaltrials.gov NCT04838795
Data collection	Participants were enrolled between 17 February and 26 March 2021, with bloods collected at baseline (prior to vaccination) and at 4, 8 and 12 weeks post vaccination.
Outcomes	Primary and secondary outcomes were outlined at clinicaltrials.gov NCT04838795.

Flow Cytometry

Plots

Confirm that:

☒ The axis labels state the marker and fluorochrome used (e.g. CD4-FITC).

☒ The axis scales are clearly visible. Include numbers along axes only for bottom left plot of group (a 'group' is an analysis of identical markers).

☒ All plots are contour plots with outliers or pseudocolor plots.

☒ A numerical value for number of cells or percentage (with statistics) is provided.

Methodology

Sample preparation	All bloods were collected into heparin tubes and processed within four hours of collection. A Ficoll-Paque (Amersham Biosciences, Little Chalfont, UK) density gradient sedimentation was used to separate the PBMCs as per manufacturer's instructions. PBMCs were cryopreserved in freezing media containing 10% DMSO in heat inactivated fetal bovine serum (FBS, ThermoFischer Scientific) and stored in liquid nitrogen.

Instrument	BD FACS Aria Fusion III flow cytometer<br>BD LSRII flow cytometer
Software	FACSDiva 9 (BD Biosciences)
Cell population abundance	Cell sorting did not form part of the methodology for this study.
Gating strategy	A figure exemplifying the gating strategy was included in the manuscript.

☒ Tick this box to confirm that a figure exemplifying the gating strategy is provided in the Supplementary Information.
